# A legacy of excellence: celebrating 40 years of the Diokno–Lapides essay contest

**DOI:** 10.1007/s11255-023-03886-6

**Published:** 2023-12-11

**Authors:** Michael Chancellor, Sarah Bartolone

**Affiliations:** Department of Urology, Oakland University William Beaumont School of Medicine, Corewell Health William Beaumont University Hospital, 3811 W 13 Mile Rd, Suite 504, Royal Oak, MI USA

**Keywords:** Neurogenic bladder, Lapides, Diokno, overactive bladder, Catheterization

## Abstract

The Diokno–Lapides Essay Contest was originally established in 1984 as the Jack Lapides Essay Contest on Urodynamic and Neurourology Research. Developed by Ananias Diokno to honor his mentor, Jack Lapides at the University of Michigan, it was funded by a grant from Marion Laboratories. The contest recognizes individuals doing outstanding work in neurourology and has been awarded yearly since 1985. Renamed the Diokno–Lapides Essay Contest in 2014, it has generated significant papers and discoveries in neurourology. Spanning 40 years, winners and other participants have attested to the contest’s influence on their careers and its opportunities for networking and mentorship across the global urology community.

## Introduction

**Fig. 1:**
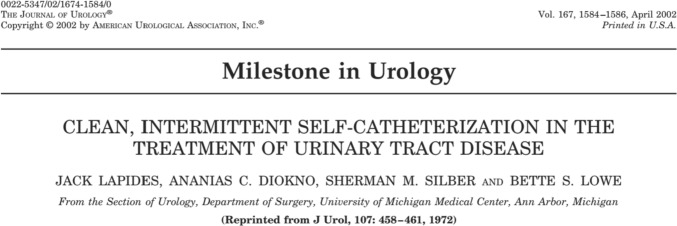
Best paper in neurology of 100 years of publication in the Journal of Urology

In 2002, The Journal of Urology commemorated its centenary by reissuing the top 100 papers published within its pages across the past century. Among this selection, a paper emerged as the pinnacle representation of neurourological impact: the 1972 publication that introduced clean intermittent catheterization to urology authored by Lapides J, Diokno AC, Silber SM, Lowe BS at the University of Michigan (UM) (Fig. 1) [1]. The landscape of clinical urology has evolved remarkably since, witnessing substantial enhancements; however, certain foundational aspects have remained resolute.

The paper titled “Clean, intermittent self-catheterization in the treatment of urinary tract disease,” published in the Journal of Urology in 1972 [1] reverberated within the urology community as a milestone and catalyst for change. Its recognition as a transformative force was swift and profound in the 1970s, and today, it continues to be the definitive benchmark in the field of neurourology. Its enduring impact persists, defining the gold standard in neurourological practice then as much as it does now.

## Contest’s founding and purpose

The Diokno–Lapides Essay Contest emerged as a homage to the exceptional contributions made by two luminaries in the field of neurourology. Driven by a desire to inspire and acknowledge budding urologists, the contest was established with the intent of nurturing academic excellence and scientific exploration. Designed as a platform for young researchers, it offers them the opportunity to showcase their work, disseminate innovative concepts, and enrich the reservoir of knowledge in the realm of neurourology.

The inaugural Diokno–Lapides Essay Contest commenced in 1984, serving as a tribute to Jack Lapides, MD upon his retirement, led by Ananias Diokno, MD. The first set of victors was celebrated during the 1985 American Urological Association annual meeting, under the stewardship of Diokno.

**Fig. 2:**
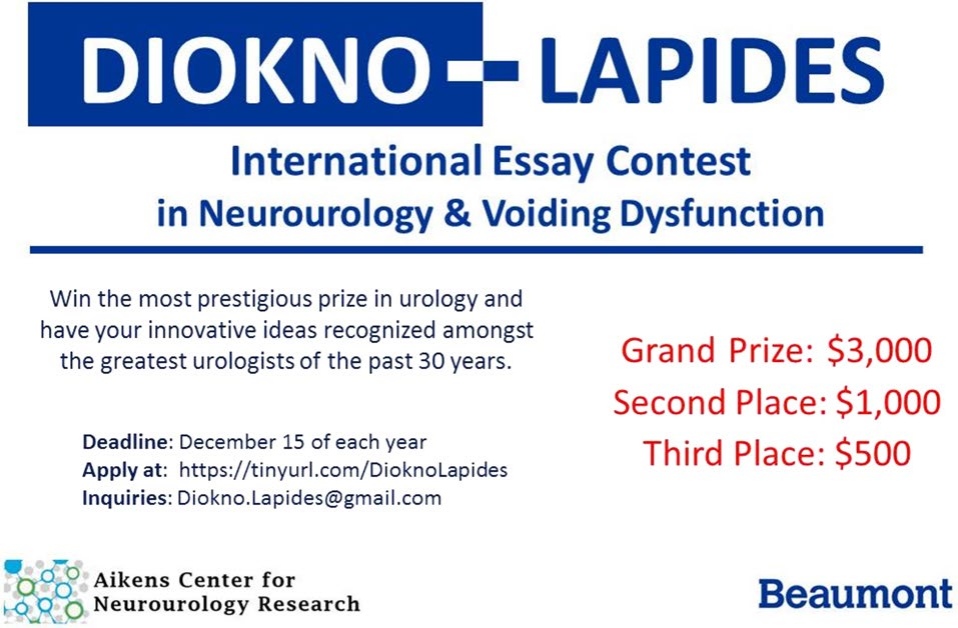
Announcement of the Diokno Lapides Award

To facilitate seamless engagement, the contest maintains an exclusive website (https://dioknolapides.wixsite.com/essay) hosting sections dedicated to contest history, essay requisites, submission procedures, and past victors. In recent times, the grand prize has amounted to $3000 US, with second and third prizes of $1000 and $500, respectively. The contest has garnered submissions from around the world, transcending geographical boundaries, particularly from Asia and Europe. In 2024, a celebratory banquet is scheduled in San Antonio for both the 2024 winners and the past grand prize recipients. The design template for the grand prize winner’s plaque is depicted in Fig. 2.

Two significant changes have been made to the award structure since its inception. The role of master steward transitioned from Diokno to Dr. Michael Chancellor in 2016. Chancellor, who completed his urology training at UM under Dr. Edward McGuire, shared a unique connection with Diokno and has been instrumental in furthering the award's legacy. Furthermore, the sponsorship of the award shifted from the pharmaceutical industry to Beaumont Health and Oakland University William Beaumont School of Medicine.

Throughout its existence, the Diokno–Lapides award has remained rooted in fairness and objectivity. Three impartial judges, devoid of conflicts of interest, are selected annually by the steward. Stripped of any identifying references, judges assess, score, and rank them. The contest encompasses both clinical and basic science aspects, with the judges being eminent figures in neurourology, spanning both medical and scientific domains. The award manager (Ms. Sarah Bartolone) compiles the final scores and announces the winners, ensuring a rigorous and transparent evaluation process.

In sum, the Diokno–Lapides Essay Contest stands as a testament to the unwavering dedication of Diokno and Lapides to fostering academic excellence in neurourology. By providing a platform for scholarly exploration and recognition, the contest encapsulates its vision and continues to shape the future of urological research.

## Jack Lapides, MD (Fig. 3)

**Fig. 3:**
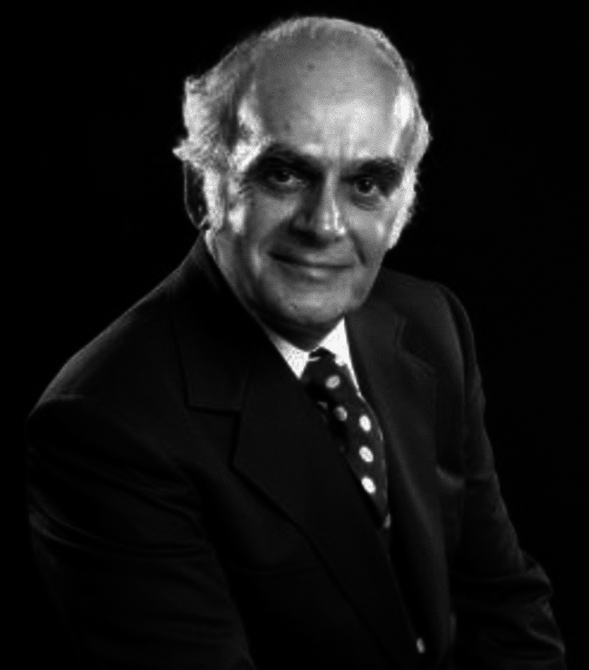
Jack Lapides, MD

Jack Lapides was born and raised in Rochester, New York and earned three degrees at UM from 1932 to 1941—a B.A, an M.A. in physiology as a Rockefeller Research fellow, and an M.D. His surgical training at UM under Frederick Coller was interrupted by service in the Army Air Corps as a flight surgeon in the Pacific Theater and he returned to Ann Arbor for another year of surgical training and then three years of urology under Reed Nesbit. Lapides then joined the faculty as an instructor and ultimately became Nesbit’s successor.

In 1968, Lapides became chief of the Section of Urology at UM, a position he held until 1983. Lapides proved to be one of the century’s most influential figures in urology. His contributions to basic and clinical research led to seminal changes in the treatment of many urological conditions [2], including the development of clean intermittent self-catheterization in the management of neurogenic bladder, an approach that ran counter to established medical practice at the time but proved to be one of the most important offerings to the field [1].

Understanding the importance of urodynamic studies in diagnosing and managing urological conditions, Lapides made significant contributions in this field as well. He developed cystometry, a technique used to assess bladder function, and the urethral pressure profile, which provides valuable information about urethral function [3]. These innovations expanded the diagnostic capabilities of urologists and facilitated more precise treatment planning.

A prominent force in academic research and scholarship, Lapides is the author of 198 scientific papers and two textbooks on urology, including Fundamentals of Urology published in 1976. He is also the author of 28 chapters in textbooks. Lapides also played an active role in various urology organizations, where he shared his knowledge and expertise through lectures and presentations. During his tenure, he received numerous honors and awards, including the prestigious Ramon Guiteras Award in 1987, and the Pediatric Urology Medal in 1989. Lapides also participated actively in national and international urologic societies.

Additionally, Lapides' dedication to education and training has nurtured generations of urologists, ensuring that his legacy lives on through their expertise and practice. In 1984, following a longstanding career as a surgeon, educator and researcher, Lapides retired from active faculty status. The UM Regents honored him with the distinction of professor emeritus of surgery. His impact on patient care, urology education, and research continues to reverberate, and his legacy serves as an inspiration for future advancements in the field.

## Ananias Diokno, MD (Fig. 4)

**Fig. 4:**
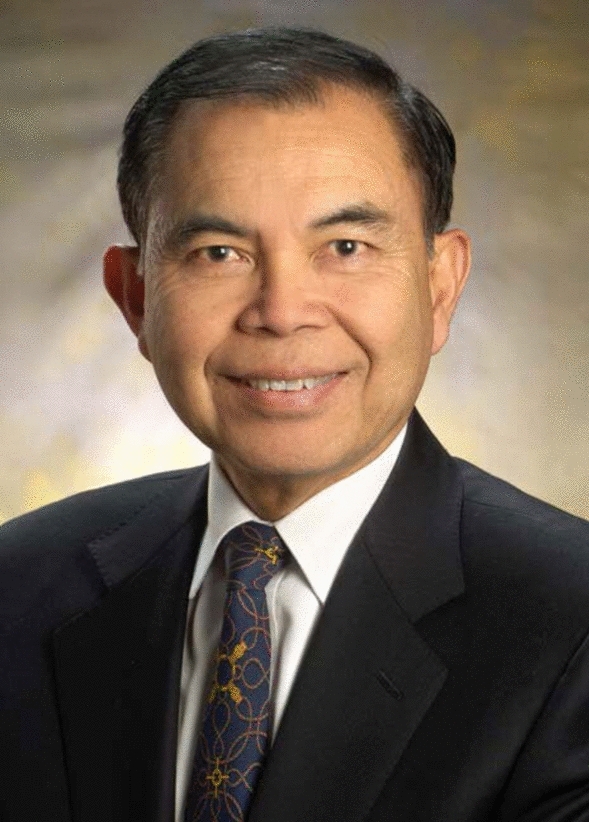
Ananias Diokno, MD

Ananias Diokno earned his medical doctorate from the University of Santo Tomas, Manila, Philippines in 1965. Diokno began training at Wayne County General Hospital, an independent training program run by Lapides. Lapides became chief of urology at UM in 1968 and enjoined the Wayne County trainees to the UM program. Following this, Diokno completed urology training under Lapides at UM in 1970 and after a year as a research fellow under Lapides joined the urology faculty. His ascent within academia was swift, attaining the position of Assistant Professor of Urology in 1972, rapidly progressing to Associate Professor in 1976, and achieving Full Professor status in 1982. In 1984, he was recruited to become the Chairman of the Department of Urology at the William Beaumont Hospital in Royal Oak, Michigan. In 2006, he ascended to the role of Executive Vice President and Chief Medical Officer of Beaumont Health System, and he played a pivotal role in the establishment of the Oakland University William Beaumont School of Medicine, where he served as a Professor of Urology.

Diokno's scholarly contributions are monumental, extending beyond his groundbreaking collaboration with Lapides. He boasts a prolific publication record comprising over 250 journal articles and book chapters, which have significantly enriched the field of urology throughout his illustrious career.

His pivotal contributions extend to the realm of geriatric urinary incontinence, where he conducted the pioneering prospective epidemiological study of urinary incontinence and lower urinary tract symptoms among community-dwelling men and women [4, 5]. Recognized with the prestigious MERIT Award by the National Institute of Aging for his consistent research excellence, he continued his exploration into the value of pelvic floor exercises in managing female urinary incontinence. His innovative combination of bladder training for frequency and urgency with pelvic floor exercises for stress incontinence led to the inception of the behavioral modification program, initially in one-on-one settings and subsequently in group sessions to educate participants about behavioral modification program.

**Fig. 5:**
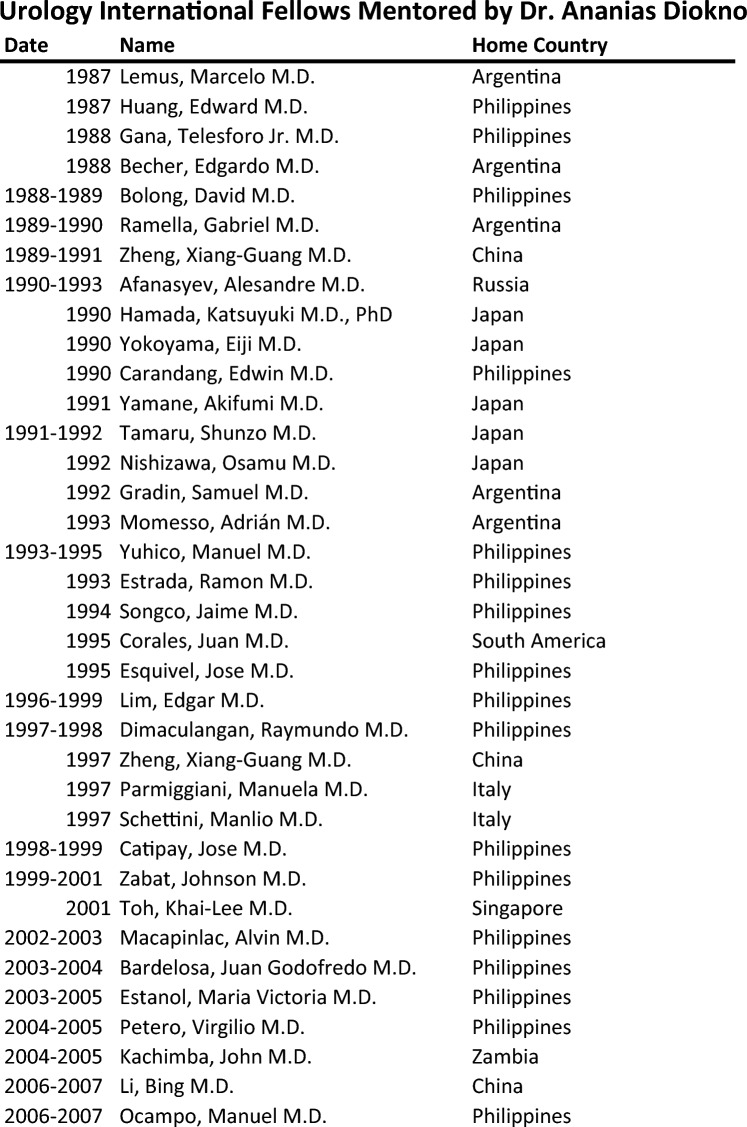
International Fellowship Dr. Ananias Diokno sponsored and mentored at William Beaumont Hospital, Royal Oak, Michigan

Beyond his research acumen, Diokno has dedicated himself to medical education and mentorship. His influence has resonated as a professor at esteemed medical institutions, nurturing the growth of the next generation of healthcare professionals. He played a pivotal role in advisory boards for significant initiatives like the first NIH KO1 training grant and executive member of the inaugural R21 research education at Beaumont entitled CURE-UAB [6]. Diokno has mentored international fellows during his tenure at Beaumont and many of whom are now serving as leaders in their country (Fig. 5).

Through his transformative research, pioneering surgical techniques, and unwavering commitment to his patients and healthcare systems, Diokno has profoundly impacted countless lives, setting new benchmarks for healthcare excellence. His enduring legacy will continue to inspire generations of medical practitioners, driving them to break barriers, foster innovation, and collectively create a healthier world for all.

## Collaborative endeavors of lapides and diokno

The Diokno–Lapides award stands as a tribute to the exceptional work of two stalwarts in neurourology, Lapides and Diokno, who made significant contributions to both Overactive Bladder (OAB) and Underactive Bladder (UAB) domains.

In the realm of Overactive Bladder (OAB), Lapides and Diokno undertook clinical research that centered on the neurophysiology and urodynamics of bladder dysfunction among spinal cord patients [7] as well as pediatric patients [8]. Their investigations encompassed pediatric urinary infections correlated with bladder dysfunction, and the complexities of pediatric enuresis and detrusor overactivity. These pioneering endeavors laid the groundwork for clinical research on oxybutynin chloride, a study that led to the FDA's approval of oxybutynin (marketed as Ditropan) for both adult and pediatric populations [9].

In the field of Underactive Bladder (UAB), the suffering experienced by individuals grappling with this condition cannot be overstated. The predominant recourse for UAB management, catheterization, brings forth a host of challenges including pain, infection risk, social discomfort, and a palpable decline in quality of life. Sadly, the treatment options and research avenues for UAB have been limited. Lapides and Diokno's instrumental work on clean self-catheterization in UAB has been transformative [1]. Furthermore, their pioneering efforts extended to drug treatments, marking an essential milestone in UAB management [10].

The legacy of Lapides’ and Diokno's contributions endured beyond their original works. Their ongoing impact is exemplified by Diokno's continued collaboration with Chancellor at Beaumont. Their shared commitment to addressing the gaps in UAB knowledge led to significant achievements. This journey encompassed writing the first comprehensive book on UAB by their pupil and mentor [11] and conducting pioneering clinical trials, notably the CURE-UAB initiative [6]. Their trailblazing endeavors continue to define the realm of underactive bladder, providing hope for those suffering from this life-altering condition.

In summary, the Diokno–Lapides award stands as a testament to the enduring contributions of Lapides and Diokno in the spheres of OAB and UAB. Their work not only transformed the landscape of urological research and practice but also kindled a beacon of hope for patients and families navigating the challenges of bladder dysfunction.

## Recognition and achievements (Table 1)

**Table 1:** List of grand prize winners of the Diokno Lapides Award over the 40 years

Year	Grand Prize	Co-GP Winner	Institution at the time of the award
2024	TBD		TBD
2023	Glenn T. Werneburg, M.D., Ph.D		Glickman Urological and Kidney Institute, Cleveland Clinic
2022	Maryrose Sullivan, Ph.D		Brigham & Women’s Hospital, Harvard Medical School
2021	Rose Khavari, M.D	Yao-Chi Chuang, M.D	Houston Methodist Hospital (Khavari); Kaohsiung Chang Gung Memorial Hospital, Taiwan (Chuang)
2020	J Todd Purves, M.D., Ph.D		Duke University School of Medicine
2019	Aaron Mickle, PhD		Washington University School of Medicine
2018	Jason Van Batavia, MD		Children's Hospital of Philadelphia
2017	Tatsuya Ihara, PhD		University of Yamanashi, Japan
2016	Tatsuya Ihara, MD, PhD		University of Yamanashi, Japan
2015	Georgi V. Petkov, PhD		University of South Carolina College of Pharmacy
2014	Kanno Yukiko, MD		Hokkaido University, Sapporo, Japan
2013	Hai-Hong Jiang, MD, PhD		Cleveland Clinic
2012	Akira Furuta, MD, PhD		Jikei University School of Medicine
2011	Takahiko Mitsui, PhD		Hokkaido University, Sapporo, Japan
2010	Pradeep Tyagi, PhD		William Beaumont Hospital, Royal Oak, MI
2009	Minoro Miyazato, MD, PhD		University of Ryukyus, Nishihara, Japan
2008	Trinity J. Bivalacqua, MD		Johns Hopkins Hospital
2007	Tetsuya Imamura, MD		Shinshu University School of Medicine
2006	Atsushi Numata, MD		Asahikawa Medical College, Japan
2005	Chuan-Guo Xiao, M.D		New York University School of Medicine
2004	Hannes Strasser, M.D		University of Innsbruck, Austria
2003	Chuan-Guo Xiao, M.D		New York University School of Medicine
2002	Karen A. Blackstone, MD		VA Medical Center, VA Boston Health Care System
2001	Satoshi Yotsuyanagi, MD		Kanazawa University School of Medicine, Japan
2000	Teruhiko Yokoyama, MD		University of Pennsylvania
1999	Naoki Yoshimura, MD, PhD	Michael B. Chancellor, MD	University of Pittsburgh Medical Center (both)
1998	Michael C. Truss, MD		Hannover Medical School, Germany
1997	Maryrose P. Sullivan, PhD	Imre Kifor, PhD	Brigham and Women's Hospital, West Roxbury VAMC, Boston
1996	Arthur L. Burnett, MD		Johns Hopkins Hospital
1995	Katsuyuki Baba, MD		University of California- San Francisco
1994	Serge Carrier, MD		University of California- San Francisco
1993	Michael B. Chancellor, MD		Jefferson Medical College
1992	Markus Hohenfellner, MD		Klinikum Barmen, Germany
1991	Jeremy W. P. Heaton, MD		Kingston General Hospital, Ontario, Canada
1990	Ellen Shapiro, MD		Children's Hospital of Wisconsin
1989	Rudd J. L. H. Bosch, MD	Hugh D. Flood, FRCSI	University of California- San Francisco (Bosch); Meath Hospital, Dublin (Flood)
1988	Barry A. Kogan, MD		University of California- San Francisco
1987	Marshall L. Stoller, MD		Tanagho
1986	K. P. Jünemann, MD		Kassel, Germany
1985	Irwin Goldstein, MD		Unknown

Table 1 provides a comprehensive record of grand prize recipients since the establishment of the award in 1984. Among these winners are prominent figures in the field, each representing exceptional contributions to world-class research. The Diokno–Lapides award has forged collaborative partnerships with both national and international organizations, underscoring its commitment to advancing neurourology education and training. Notably, a key element of the award's significance lies in its pivotal role in nurturing the progression of education and training within the realm of neurourology.

## Impact of the Diokno–Lapides essay contest

The Diokno–Lapides Essay Contest has received recognition and accolades within the field of urology. Its commitment to academic excellence and scientific rigor has garnered attention from prestigious urological societies, institutions, and organizations. The contest has been supported by prominent medical societies, serving as a platform for collaboration between academia and industry. Furthermore, the contest has established partnerships with other organizations in the urology community to enhance its impact. Collaborations with medical journals and academic institutions have facilitated the dissemination of winning essays, showcasing the contest's role in disseminating valuable research findings.

The Diokno–Lapides Essay Contest has made significant contributions to urology research and knowledge. Numerous papers and discoveries originating from the contest have shed light on critical aspects of urological conditions, diagnostic techniques, treatment modalities, and patient outcomes. These findings have advanced the field by providing novel insights, refining existing practices, and influencing clinical guidelines.

Beyond scientific contributions, the contest has had a profound impact on participants. Winners and finalists have acknowledged the contest's role in shaping their careers and fostering mentorship opportunities. The recognition associated with the Diokno–Lapides Essay Contest has opened doors for collaboration, funding opportunities, and career advancement within the urology community.

## Challenges and future directions

While the Diokno–Lapides Essay Contest has achieved significant success, it has also faced challenges along its journey. As the contest attracts more talented participants, maintaining a fair and rigorous evaluation process becomes crucial. Striking a balance between recognizing excellence and ensuring inclusivity remains a challenge.

Another challenge lies in adapting to the evolving landscape of scientific communication. With the rapid advancement of technology and the emergence of new platforms for research dissemination, the contest must continue to embrace innovative formats and modes of presentation. This includes exploring multimedia submissions, interactive displays, or virtual platforms to enhance the impact and accessibility of the contest.

To overcome these challenges and ensure the future success of the Diokno–Lapides Essay Contest, several strategies can be considered. First, maintaining close collaboration with leading urological societies and institutions can help attract top talent and secure ongoing support. Second, engaging with participants through mentorship programs, workshops, and networking events can nurture the development of young researchers and foster a sense of community within the contest.

## Conclusion

The Diokno–Lapides Essay Contest has played a vital role in advancing neurourology research and education. Through its origins, evolution, impact, recognition, and achievements, the contest has contributed to the body of knowledge in the field and inspired generations of urologists.

As we reflect on the history and accomplishments of the Diokno–Lapides Essay Contest, it is evident that ongoing support, collaboration, and a commitment to scientific excellence are crucial for its sustained success. By nurturing talent and embracing new approaches to research dissemination, the contest can continue to inspire and drive advancements in the field of urology for years to come.

## Data Availability

Data is fully available at the Diokno Lapides Essay website listed in this article.
